# National Medical Teams during European Athletics Championships from 2009 To 2024: Composition, Gender Distribution, and Influence on Team Performance

**DOI:** 10.1186/s40798-025-00864-3

**Published:** 2025-05-15

**Authors:** Pascal Edouard, Spyridon Iatropoulos, Karolina Velebova, Ciara McCallion, Marianna Kiss, Pierre-Eddy Dandrieux, Pedro Branco, Jane Thornton, Karsten Hollander

**Affiliations:** 1 Inter-university Laboratory of Human Movement Science (LIBM EA 7424), University of Lyon, University Jean Monnet, Saint Etienne, F-42023 France; 2 Department of Clinical and Exercise Physiology, Sports Medicine Unit, Faculty of Medicine, IRMIS, Campus Santé, University Hospital of Saint-Etienne, Saint-Etienne, cedex 2 42 055 France; 3 European Athletics Medical & Anti Doping Commission, European Athletics Association (EAA), Lausanne, Switzerland; 4 Body Solution Clinic, Czech Track and Field Association, Czech Olympic Team, Prague, Czechia; 5 Athletics Ireland, Northwood Court, Northwood Business Campus, Satrym Dublin, Ireland; 6 National Institute for Sports Medicine, Budapest, Hungary; 7 Mines Saint-Etienne, Univ Lyon, Univ Jean Monnet, Centre CIS, Saint-Etienne, F-42023 France; 8 Medical and Scientific Department, International Olympic Committee, Lausanne, Switzerland; 9 Western Centre for Public Health & Family Medicine, Schulich School of Medicine & Dentistry, Western University, London, ON Canada; 10 Institute of Interdisciplinary Exercise Science and Sports Medicine, MSH Medical School Hamburg, Hamburg, Germany

**Keywords:** Medical coverage, Medical team, Surveillance, Epidemiology, Track and field, Top-level athletes

## Abstract

**Background:**

Having the overall goal to help countries/teams in the preparation of their national medical teams for international athletics championships, we aimed to describe the composition of national medical teams, including gender distribution, and to explore its potential association with team performance, during European Athletics championships. We conducted a retrospective study covering 15 consecutive outdoor and indoor European Athletics championships between 2009 and 2024 including the national medical team members and athletes registered. We extracted the number of national medical team members by profession and gender, the ratio of athletes per national medical team member, and the number of medals per athlete. Potential associations were explored using Spearman’s correlations.

**Results:**

During the 15 consecutive European Athletics championships between 2009 and 2024, 54 European Athletics member federations participated at one or more of the championships, corresponding to 726 country-participations, from which 68.5% had a national medical team. The national medical team included: 71.0% physiotherapists and 29.0% physicians, 20.7% women and 79.3% men. There was a median of 11 (range: 1–43) athletes per physiotherapist and 23 (range: 3–64) athletes per physician. There was a small but significant negative correlation between the number of medals per athlete and the ratio of athletes per medical team member (*r*=-0.33; *p* < 0.001).

**Conclusions:**

During the European Athletics championships, approximately two-thirds of countries/teams had a national medical team, with a median of eight athletes per medical team member, with large variation between teams. Only one out of five medical team members were women. When the number of athletes per medical team member was higher, this was associated with a lower number of medals per athlete. These findings may be of help to assemble effective and successful medical teams in future championships.

**Supplementary Information:**

The online version contains supplementary material available at 10.1186/s40798-025-00864-3.

## Background

International athletics championships typically represent the pinnacle of an athletics season. Participating in these championships is associated with a risk of sustaining or exacerbating injury and/or illness [1, 2]. In addition, athletes can participate with chronic injuries and/or illnesses [3]. To manage potential health problems, as the guarantor of athletes’ health, many countries/teams assemble a national medical team for international athletics championships [4]. 

There is currently, to our knowledge, no clear recommendation on the composition of the national medical teams, in athletics or in any other sport. What is the optimal allocation strategy of human resources (e.g., physicians, physiotherapists, other health professionals) for a given team or number of athletes? Such decisions are likely made with the experience of team leaders or health professionals, and/or under the constraint of the available budget. It could be also determined/influenced, for instance, by the number of athletes going to the championships, the qualification of physicians for sports medicine, and/or the general country resources. Hence, a description of current practice, an analysis of country-related characteristics that could play a role in the national medical team size and composition, and of potential associations between national medical team composition and team performance during championships could assist countries/teams in planning their national medical teams for future international athletics championships.

In addition, medical team composition in terms of gender distribution is also of interest. Indeed, athletes may be more likely to consult health professionals of a specific gender for specific health problems, for instance, women physicians are preferred to deal with sexual health, diet, and mental health [5–7]. Women health professionals may be more competent and appropriate to manage several specific women’s health issues (e.g., gynaecological issues) [7]. Thus, gender parity seems important for athlete’s health management. However, gender disparities were reported in medical teams with large imbalance between the number of women versus men among medical teams [8], for instance in the National Collegiate Athletic Association (approximately 10% of women team physicians) [9, 10] and the Men’s and Women’s National Basketball Association (2.4% and 28.6% of women team physicians) [11]. In this context, it is relevant to also explore the gender distribution in national medical teams during international athletics championships.

Having the overall goal to help countries/teams in the preparation of their national medical teams for international athletics championships, we aimed (1) to describe the size and composition of national medical teams, especially regarding profession and gender distribution, (2) to analyse potential country-related factors that may be associated with national medical team composition, and (3) to explore potential associations between national medical teams’ composition and team performance, during European Athletics championships.

## Methods

### Study Design

We conducted a retrospective study in the context of 15 consecutive outdoor and indoor European Athletics championships that took place between 2009 and 2024: European Outdoor Championships (EOC) 2010, 2012 [12], 2014, 2016, 2018 [13], 2022 [14, 15], and 2024 [16], and European Indoor Championships (EIC) 2009, 2011 [17], 2013 [18], 2015, 2017, 2019, 2021 and 2023. The study protocol was reviewed and approved by the Saint-Etienne University Hospital Ethics Committee (Institutional Review Board: IORG0007394; IRBN742020/CHUSTE).

### Population

The participants were all national medical team members and athletes registered at one or more of the 15 European championships.

### Data Collection

National medical team members’ and athletes’ data were extracted from the lists of registered medical teams and athletes provided by European Athletics [19]. For each championship and each country, we extracted (i) the number of registered women and men of national medical teams and their profession (physiotherapists or physicians; no more details were provided regarding medical speciality for physicians, and if physiotherapists were recognised by the profession, or they were other therapists (e.g., chiropractor, soft tissue therapist, osteopath)) from the list of registered medical teams, and (ii) the number of registered women and men athletes from the list of registered athletes. The gender of medical team members and athletes was reported by the individuals themselves during the registration process at the championship.

We then calculated the following:

i) total number of national medical team members (i.e., national medical team size);
ii) total number of registered athletes (i.e., athletes’ team size);
iii) gender disparity, defined as *(number of men– number of women) / (total number)*, for both medical national teams and athletes;
iv) profession disparity, defined as *(number of physiotherapists– number of physicians) / (total number)*, only for national medical teams;
v) ratio of registered athletes per medical team member (i.e., number of athletes divided by medical team size);
vi) ratio of athletes per team physiotherapist, and per team physician;
vii) ratio of women athletes per woman medical team member, and ratio of men athletes per man medical team member.

Regarding country-related characteristics that could influence the composition of national medical teams, we collected for each country and each championship year: (i) the total population [20], (ii) the gross domestic product per capita (GDP) in US dollars ($) [20], and (iii) the status of sports medicine speciality (i.e., whether there was a full, or partial speciality programme recognised in a given country at a given year or no speciality programme at all) by using information from Pigozzi [21], Neunhaeuserer et al., [22] and updated by direct communication with sports and exercise medicine physicians from countries with missing information.

For team performance, we collected for each championship and each country, the total number of medals, according to the official results derived from the dedicated page of each championship in Wikipedia [23]. We then calculated the number of medals per registered athlete.

### Statistical Analyses

We performed a descriptive analysis of the collected data, using frequencies with percentages for categorical variables, and median (interquartile range (IQR)) for continuous variables.

For the description of the national medical teams’ composition, we also compared (i) the proportion of countries/teams with and without national medical teams according to cut-off of number of athletes per country/team and (ii) the proportion of women between physiotherapists and physicians, using the Chi-2 test of independence. We also compared the national medical team size and the ratio of athletes per medical team member between outdoor and indoor championships using the Mann-Whitney U test.

Regarding the potential country-related characteristics that could play a role in national medical teams composition, we analysed the potential associations between each one of the (i) medical team size, (ii) ratio of athletes per medical team member, (iii) profession disparity, and (iv) gender disparity, and each one of the (a) athletes’ team size, (b) country’s population, (c) country’s GDP, and (d) sports medicine specialty status, using Spearman’s correlations. Only cases with at least one medical team member were included in this analysis.

Regarding national medical team composition and team performance, we analysed the potential associations between the number of medals per athlete and each one of the (a) ratio of athletes per medical team member, (b) profession disparity, and (c) gender disparity, using Spearman’s correlations. Only cases with at least one medical team member were included in this analysis.

The significance level was initially set at *P* < 0.001 to adjust for multiple comparisons. Statistical analyses were performed using ‘Statsmodels’ package in Python (https://www.python.org) [24]. 

## Results

### Population

During the 15 European Athletics championships that took place during the 16-year period from 2009 to 2024, 54 European Athletics member federations participated at one or more of the championships, corresponding to 726 country/team-participations including a total of 15,289 registered athletes (Table 1).

**Table 1:** Number of countries/teams, registered athletes, National medical teams, registered team physicians and physiotherapists during the 15 European athletics championships 2009–2024. Gender disparity was calculated as: *(number of men– number of women) / (total number).* Positive values indicate more men than women and negative values indicate more women than men. Zero would indicate equal number of men and women. There were 726 country/team-participations: 39 (72.2%) countries/teams participated in all 15 championships, 4 (7.4%) in 14 championships, 1 (1.9%) in 13 championships, 4 (7.4%) in 12 championships, 2 (3.7%) in 7 championships, 1 (1.9%) in 4 championships, 1 (1.9%) in 3 championships, 1 (1.9%) in 2 championships, and 1 (1.9%) in 1 championship

	Country participations	Total registered athletes	Registered men athletes (*n* (%))		Registered women athletes (*n* (%))		Registered national medical teams (*n* (%))		Registered physicians and physiotherapists	Registered physicians (*n* (%))		Registered physiotherapists (*n* (%))		Registered physicians and physiotherapists men (*n* (%))		Registered physicians and physiotherapists women (*n* (%))		Gender disparity (median [IQR])	
**Indoor**	**379**	**4905**	**2641**	**(53.8)**	**2264**	**(46.2)**	**248**	**(65.4)**	**612**	**176**	**(28.8)**	**436**	**(71.2)**	**496**	**(81.0)**	**116**	**(19.0)**	**1.0**	**[0.33–1.0]**
EIC2009	45	568	316	(55.6)	252	(44.4)	31	(68.9)	73	24	(32.9)	49	(67.1)	59	(80.8)	14	(19.2)	1.0	[0.27–1.0]
EIC2011	46	593	324	(54.6)	269	(45.4)	32	(69.6)	80	23	**(28.8)**	57	(71.3)	68	(85.0)	12	(15.0)	1.0	[0.33–1.0]
EIC2013	47	577	320	(55.5)	257	(44.5)	28	(59.6)	66	21	(31.8)	45	(68.2)	58	(87.9)	8	(12.1)	1.0	[0.6–1.0]
EIC2015	49	643	363	(56.5)	280	(43.5)	34	(69.4)	82	25	(30.5)	57	(69.5)	71	(86.6)	11	(13.4)	1.0	[0.62–1.0]
EIC2017	49	561	282	(50.3)	279	(49.7)	33	(67.3)	82	23	(28.0)	59	(72.0)	68	(82.9)	14	(17.1)	1.0	[0.33–1.0]
EIC2019	49	637	330	(51.8)	307	(48.2)	36	(73.5)	89	25	(28.1)	64	(71.9)	69	(77.5)	20	(22.5)	0.83	[0.33–1.0]
EIC2021	47	733	405	(55.3)	328	(44.7)	25	(53.2)	66	15	(22.7)	51	(77.3)	50	(75.8)	16	(24.2)	0.6	[0.33–1.0]
EIC2023	47	593	301	(50.8)	292	(49.2)	29	(61.7)	74	20	(27.0)	54	(73.0)	53	(71.6)	21	(28.4)	1.0	[0–1.0]
**Outdoor**	**347**	**10,384**	**5523**	**(53.2)**	**4861**	**(46.8)**	**249**	**(71.8)**	**1114**	**324**	**(29.1)**	**790**	**(70.9)**	**873**	**(78.4)**	**241**	**(21.6)**	**0.6**	**[0.33–1.0]**
EOC2010	50	1371	762	(55.6)	609	(44.4)	35	(70.0)	150	52	(34.7)	98	(65.3)	123	(82.0)	27	(18.0)	0.67	[0.38–1.0]
EOC2012	50	1352	745	(55.1)	607	(44.9)	36	(72.0)	138	42	(30.4)	96	(69.6)	114	(82.6)	24	(17.4)	0.69	[0.33–1.0]
EOC2014	50	1439	787	(54.7)	652	(45.3)	34	(68.0)	149	45	(30.2)	104	(69.8)	127	(85.2)	22	(14.8)	1.0	[0.5–1.0]
EOC2016	50	1469	750	(51.1)	719	(48.9)	36	(72.0)	152	45	(29.6)	107	(70.4)	122	(80.3)	30	(19.7)	0.56	[0.33–1.0]
EOC2018	51	1570	828	(52.7)	742	(47.3)	39	(76.5)	183	52	(28.4)	131	(71.6)	143	(78.1)	40	(21.9)	0.54	[0.33–1.0]
EOC2022	48	1540	805	(52.3)	735	(47.7)	33	(68.8)	164	41	(25.0)	123	(75.0)	116	(70.7)	48	(29.3)	0.50	[0.2–1.0]
EOC2024	48	1643	846	(51.5)	797	(48.5)	36	(75.0)	178	47	(26.4)	131	(73.6)	128	(71.9)	50	(28.1)	0.52	[0.33–1.0]
**Total Indoor + Outdoor**	**726**	**15,289**	**8164**	**(53.4)**	**7125**	**(46.6)**	**497**	**(68.5)**	**1726**	**500**	**(29.0)**	**1226**	**(71.0)**	**1369**	**(79.3)**	**357**	**(20.7)**	**0.80**	**[0.3–1.0]**

### National Medical Team Composition

Among the 726 country-participations, 68.5% had a national medical team, with variations from 53.2 to 76.5% according to European Athletics championships (Table 1). All countries/teams with more than 10 registered athletes had a national medical team except in 5 cases, and countries/teams without any medical team had a small number of registered athletes (Table 2). The national medical team size was significantly higher for outdoor than indoor championships (*p* < 0.001), but the ratio of athletes per medical team member was also significantly higher for outdoor than indoor championships (*p* < 0.001) (Table 3).

**Table 2:** Number of countries with and without medical teams according to the number of registered athletes in the countries during the 15 European athletics championships 2009–2024. There was a significant difference in the distribution of countries/teams with and without National medical teams according to the cut-off of number of athletes per country/team participation (Chi2 = 228.8; *p* < 0.001)

Number of athletes per country participation	Number of countries	Number of countries without medical teams (*n* (%))		Number of countries with medical teams (*n* (%))		Number of countries with at least one team physiotherapist (*n* (%))		Number of countries with at least one team physician (*n* (%))		Number of countries with at least one team physiotherapist & one team physician (*n* (%))	
**> 100**	15	0	(0.0)	15	(100.0)	15	(100.0)	15	(100.0)	15	(100.0)
**76–100**	21	0	(0.0)	21	(100.0)	21	(100.0)	21	(100.0)	21	(100.0)
**51–75**	44	0	(0.0)	44	(100.0)	44	(100.0)	43	(97.7)	43	(97.7)
**26–50**	134	1	(0.0)	133	(99.3)	133	(100.0)	118	(89.5)	118	(89.5)
**16–25**	89	1	(1.1)	88	(98.9)	85	(96.6)	62	(70.5)	59	(67.0)
**11–15**	91	3	(3.3)	88	(96.7)	85	(96.6)	52	(59.1)	49	(55.7)
**6–10**	92	22	(23.9)	70	(76.1)	66	(94.3)	26	(37.1)	22	(31.4)
**< 6**	240	202	(84.2)	38	(15.8)	34	(89.5)	5	(13.2)	1	(2.6)
**Total**	726	229	(31.5)	497	(68.5)	483	(97.2)	342	(68.8)	328	(66.0)

**Table 3:** Number of registered physicians and physiotherapists per medical team, and ratio of registered athletes by the number of health professionals (i.e., number of athletes divided by number of total National medical team members, by number of physiotherapists, by number of physicians), during the 15 European athletics championships 2009–2024

	Registered physicians and physiotherapists per medical teams				Registered physiotherapists per medical teams				Registered physicians per medical teams				Ratio of the number of athletes divided by the number of physicians and physiotherapists per medical teams				Ratio of the number of athletes divided by the number of physiotherapists per medical teams				Ratio of the number of athletes divided by the number of physicians per medical teams			
	Median	[IQR]	Min	Max	Median	[IQR]	Min	Max	Median	[IQR]	Min	Max	Median	[IQR]	Min	Max	Median	[IQR]	Min	Max	Median	[IQR]	Min	Max
**Indoor**	2	**[1–3]**	**1**	**7**	**1**	**[1–2]**	**0**	**5**	1	**[0–1]**	**0**	**3**	**7**	**[5.5–9]**	**1.0**	**31.0**	**9.55**	**[7–12.46]**	**1.0**	**31.0**	**17**	**[12–25.75]**	**3.0**	**53.0**
EIC2009	2	[1–3]	1	6	1	[1–2]	0	4	1	**[0–1]**	0	2	7	[6–8.17]	2.3	20.0	12	[7–13.33]	3.0	20.0	15	[12.75–20]	6.0	37.0
EIC2011	2	[1–3]	1	7	1.5	[1–2]	0	5	1	**[0–1]**	0	3	6.56	[5.25–9.08]	3.3	12.5	8.5	[7–11.5]	3.7	25.0	16	[12–23.38]	8.0	40.0
EIC2013	2	[1–3]	1	5	1	[1–2]	0	4	1	**[0–1]**	0	2	6.63	[6–9]	1.7	13.0	10.25	[7.12–12]	2.5	18.5	17.25	[12–24.62]	4.0	37.0
EIC2015	2	[1–3]	1	6	1	[1–2]	0	5	1	**[0–1]**	0	2	7.75	[5.75–9]	3.0	12.0	10.25	[8–12]	4.0	16.0	18.75	[11.75–24]	3.0	38.0
EIC2017	2	[1–3]	1	6	2	[1–2]	1	5	1	**[0–1]**	0	2	6	[4.33–7.5]	1.0	11.0	8	[6–11.33]	1.0	16.0	15	[11.5–27.5]	8.0	34.0
EIC2019	2	[1–3]	1	7	1	[1–2]	0	5	1	**[0–1]**	0	2	6	[5–8.5]	1.0	15.0	9	[6–11.5]	1.0	19.0	16	[13–24]	6.0	53.0
EIC2021	2	[1–4]	1	6	2	[1–3]	1	4	0	**[0–1]**	0	3	9.8	[8–13]	5.6	31.0	12.33	[9–16]	7.0	31.0	30	[25.75–38.75]	14.7	49.0
EIC2023	2	[1–4]	1	7	2	[1–3]	0	5	1	**[0–1]**	0	2	8	[5.67–9.5]	3.0	14.0	10.25	[7.75–11]	3.0	21.0	16.5	[13.5–26.75]	5.0	42.0
**Outdoor**	**4**	**[2–6]**	**1**	**21**	**3**	**[1–4]**	**0**	**15**	**1**	**[1–2]**	**0**	**6**	**8.75**	**[7–11]**	**1.0**	**25.0**	**12**	**[9.67–15.67]**	**1.0**	**43.0**	**27**	**[21–36]**	**4.0**	**64.0**
EOC2010	4	[2-5.5]	1	10	2	[1–4]	0	7	1	[1–2]	0	4	8.4	[7.42–10.38]	3.0	16.0	12.75	[10.54–17.94]	3.0	23.0	23.25	[18.5–32.25]	12.0	43.0
EOC2012	3	[2–5]	1	10	2	[1-3.25]	0	8	1	[1–2]	0	3	9	[6.75–10.72]	3.3	25.0	12	[10.12–14.22]	4.0	26.0	28	[23–35.25]	10.0	54.0
EOC2014	3	[2.25-6]	1	15	3	[2–4]	1	11	1	[1–2]	0	4	9.83	[7.75–12.48]	3.7	17.5	14	[10.08–17.38]	4.0	30.0	27.5	[23–39.38]	10.8	62.0
EOC2016	3.5	[2-5.25]	1	15	2	[1.75-4]	0	11	1	[1–2]	0	4	8.47	[7.33–11.69]	2.0	25.0	12.38	[10.17–16.75]	2.0	25.0	26.75	[19–41.88]	4.0	64.0
EOC2018	4	[2–6]	1	21	3	[1-4.5]	1	15	1	[0.5-2]	0	6	8.75	[6.37–9.7]	1.0	21.5	11	[9 -14.92]	1.0	43.0	27	[22–35]	12.0	55.5
EOC2022	3	[2–6]	1	17	3	[2–4]	1	14	1	[0–2]	0	5	9	[7–11.5]	2.0	21.0	12.71	[9.36–15]	2.0	27.0	28.83	[19–45.25]	8.0	62.0
EOC2024	3.5	[2–6]	1	15	3	[2–5]	1	10	1	[0–2]	0	5	8.83	[6–10.82]	2.0	19.0	12	[9.46–14.79]	2.0	19.0	26.5	[17.5–35.67]	10.0	63.0
**Total**	**3**	**[2–5]**	**1**	**21**	**2**	**[1–3]**	**0**	**15**	**1**	**[0–1]**	**0**	**6**	**8**	**[6–10]**	**1.0**	**31.0**	**11**	**[8–14.3]**	**1.0**	**43.0**	**23**	**[15–33]**	**3.0**	**64.0**

#### Profession Distribution

Included in these national medical teams were a total of 1,726 registered national medical team members: 1,226 (71.0%) physiotherapists and 500 (29.0%) physicians (Table 1). When a country had a national medical team, there was in almost all cases (97.2%) at least one physiotherapist (Table 2). The number of national medical team members per country and per championship varied from 1 to 21 (Table 3). There were 2 [IQR: 1–3] physiotherapists and 1 [IQR: 0–1] physicians per national medical team (Table 3). The median profession disparity was 0.4 [IQR: 0.2-1] (range: -1 to 1), meaning that there were 2.3 physiotherapists per 1 physician.

#### Number of Athletes Per National Medical Team Members

There were 8 [IQR: 6–10] athletes per national medical team member (range: 1–31) (Table 3). According to the profession, there were 11 [IQR: 8-14.3] (range: 1 to 43) athletes per physiotherapist and 23 [IQR: 15–33] (range: 3 to 64) athletes per physician (Table 3).

#### Gender Distribution

Among the 1,726 registered national medical team members, 357 (20.7%) were women and 1,369 (79.3%) were men (Table 1). The median gender disparity was 0.8 [0.3-1] (Table 1), corresponding to 1 woman for every 9 men medical team members. Half of the countries/teams (49.5%; 246 out of the 497 country-participations) had no woman medical team member. Nineteen (3.8%) countries/teams had only women medical team members. The proportion of women was higher among physicians (24.4%) than physiotherapists (19.2%) (Chi2 = 5.93; *p* = 0.015). When there was a woman medical team member, there were 11 [6.8–18] (range: 0 to 60) women athletes per woman medical team member, and when there was a man medical team member, there were 5 [3.5-7] (range 1 to 32) men athletes per man medical team member (Table 4).

**Table 4:** Ratio of registered athletes by the number of physiotherapists and physicians according to gender during the 15 European athletics championships 2009–2024

	Number of women per medical team				Number of men per medical team				Ratio of the number of women athletes divided by the number of women medical team members				Ratio of the number of men athletes divided by the number of men medical team members			
	median	[IQR]	Minimal	Maximal	median	[IQR]	Minimal	Maximal	median	[IQR]	Minimal	Maximal	median	[IQR]	Minimal	Maximal
**Indoor**	**0**	**[0–1]**	**0**	**3**	**2**	**[1–3]**	**0**	**7**	**8**	**[5–13]**	**0**	**29**	**4.5**	**[3–6]**	**1**	**17**
EIC2009	0	[0–1]	0	2	1	[1–2]	0	6	6	[4–11]	1	17	4.5	[3–6]	1	13
EIC2011	0	[0–1]	0	2	2	[1–2]	0	7	5	[2.25–6]	1	15	4.42	[3–5]	1	10
EIC2013	0	[0–1]	0	1	2	[1–2.25]	1	5	7.5	[5–11.5]	0	19	4.12	[3–5.12]	1	9.5
EIC2015	0	[0–1]	0	1	2	[1–3]	0	5	6	[3–15.5]	1	19	4.7	[3.42–6]	1	12
EIC2017	0	[0–1]	0	2	2	[1–3]	0	5	8	[6–12]	2	20	3.5	[2.38–8]	1	8
EIC2019	0.5	[0–1]	0	2	1.5	[1–2]	0	5	8	[6.62–12.38]	0	29	4.1	[2.5–5]	1	11
EIC2021	1	[0–1]	0	3	2	[1–3]	1	4	12	[9–15]	6	26	6.67	[5.25–11]	2	17
EIC2023	0	[0–1]	0	3	1	[1–3]	0	5	8	[4.33–10]	2	17	4.58	[3.62–6]	1	15
**Outdoor**	**1**	**[0–1]**	**0**	**5**	**3**	**[2–5]**	**0**	**17**	**14**	**[9–21]**	**0**	**60**	**5.69**	**[4.13–8]**	**1**	**32**
EOC2010	1	[0–1]	0	3	3	[2–5]	1	10	13.25	[7.5–18.75]	3	60	6	[4.1–7.56]	3	15
EOC2012	1	[0–1]	0	3	3	[1–4.25]	0	9	12.5	[6.12–20.5]	1	51	6.33	[4.18–8.29]	3	17
EOC2014	0	[0–1]	0	4	3	[2–5]	1	11	13	[12–23.25]	10	36	5.5	[4.48–8.38]	2	15
EOC2016	1	[0–1]	0	3	3	[1.75–4]	1	12	18	[10.75–23.75]	6	46	5.33	[4–7.85]	1	25
EOC2018	1	[0–1]	0	4	3	[1–4.5]	0	17	12	[9–18]	0	45	5.33	[4–7.29]	2	32
EOC2022	1	[0–2]	0	5	3	[2–4]	0	13	13	[8.4–23.5]	2	40	6.5	[5–9.12]	1	15
EOC2024	1	[0–3]	0	5	2	[2–4.25]	1	11	15.17	[8.81–19.5]	4	37	6.1	[4.38–8.14]	1	15
**Total**	**1**	**[0–1]**	**0**	**5**	**2**	**[1–4]**	**0**	**17**	**11**	**[6.8–18]**	**0**	**60**	**5**	**[3.5–7]**	**1**	**32**

### Country-Related Characteristics Associated with National Medical Team Composition

There were positive significant correlations between (i) the national medical team size and the number of registered athletes (*r* = 0.89; *p* < 0.001) (Supplementary Fig. 2), (ii) the national medical team size and the total country population (*r* = 0.70; *p* < 0.001), (iii) the ratio of athletes per medical team member and the number of registered athletes (*r* = 0.54; *p* < 0.001), (iv) the ratio of athletes per medical team member and the total country population (*r* = 0.23; *p* < 0.001), and (v) the profession disparity and the GDP (*r* = 0.38; *p* < 0.001), and there were negative significant correlations between (vi) the profession disparity and the number of registered athletes (*r*=-0.26; *p* < 0.001), (vii) the profession disparity and the sports medicine specialty status (*r*=-0.30; *p* < 0.001), and (viii) the gender disparity and the GDP (*r*=-0.28; *p* < 0.001) (Supplementary Table 5).

### Associations between National Medical Team Composition and Team Performance

**Fig. 1:**
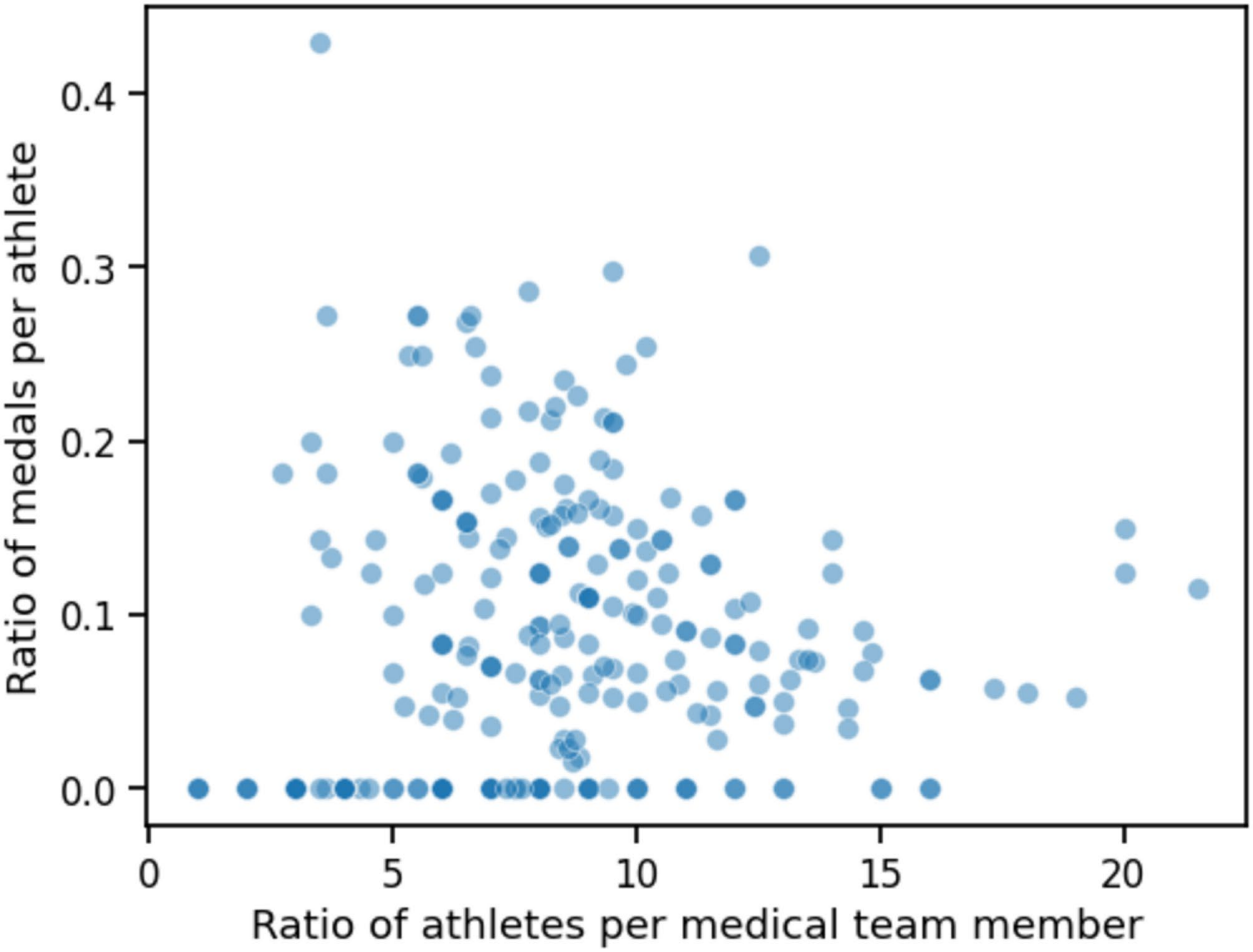
Associations between national medical team composition and team performance showing a significant correlation between the number of medals per athlete and the ratio of athletes per medical team member (Spearman’s *r*=-0.33; *p* < 0.001)

There was a significant negative correlation between the number of medals per athlete and the ratio of athletes per medical team member (*r*=-0.33; *p* < 0.001) (Fig. 1 and Supplementary Table 6).

## Discussion

The main findings of the present study were that, during European Athletics championships, (1) 68.5% of countries/teams had a national medical team, and among countries/teams with more than 10 registered athletes, 98.7% had a medical team, (2) according to the median values, there were 8 athletes per medical team member, and more specifically 11 athletes per physiotherapist and 23 athletes per physician, with large variation among countries/teams, (3) on average 1 out of 5 medical team members were women but according to the teams’ median, there was 1 woman for every 9 men medical team members, (4) several aspects of the national medical team composition (size, ratio of athletes per medical team member, profession and gender disparity) were associated with country-related characteristics (team size, population size, GDP and sports medicine specialty status), and (5) having more athletes per national medical team member was correlated with fewer medals per athlete.

### An Overview of the National Medical Team Composition

The present study provides a descriptive analysis of the composition of national medical teams during the European Athletics Championships, which is, to our knowledge, the first of its kind. We believe that this can be of interest to the sports and exercise medicine community, especially since no clear recommendation exists to determine the composition of the medical teams, in athletics and in other sports.

This study can neither determine if the reported compositions were appropriate nor optimal to protect athletes’ health. However, this study provided a valuable description of the current situation, which could aid in better preparation for future championships. The medical team size was correlated to the athletes’ team size (supplementary Fig. 2). This result can of course be intuitive: more athletes could explain more medical team members. Then, the median ratio of athletes per medical team member was eight athletes per medical team member. The ratio of athletes per medical team member was correlated with the number of registered athletes per team. We could interpret that as the number of athletes was not linearly associated with the number of medical team members: the bigger the team, the fewer medical team members were added.

Regarding the ratio of athletes per medical team member, there were differences according to profession: 11 (range: 1–43) athletes per physiotherapist, and 23 (range: 3–64) athletes per physician. Our results showed that national medical teams included approximately 2–3 physiotherapists for every 1 physician, again with large variation between countries/teams, with the minimal national medical team member being a physiotherapist. Bigger teams showed less profession disparity, which could be interpreted as the role of physician was increasingly more important in teams of larger size. The country’s status regarding the recognition and organisation of the sports medicine specialty was also associated with greater presence of physicians in the team. This can be explained by the fact that countries recognising sports medicine as a specialty may have an overall better understanding of the need for physicians in the medical teams of international championships, and/or that physicians with a recognized specialty of sports have undergone formal/regulated training within sports.

Finally, the type of championships was also associated with the medical team size. The medical team sizes were higher for outdoor versus indoor championships. Such a result could be linked to the number of events and rounds during outdoor championships, which consequently lead to more athletes registered at outdoor championships, and bigger national teams than during indoor championships. In addition, the championship duration could play a role, since typically indoor championships are shorter than outdoor championships. However, the ratio of athletes per medical team member was lower in indoor than outdoor championships. This means that in indoor championships, teams were smaller but with more medical team members per athlete, compared to outdoor championships.

### Extensive Gender Imbalance in the National Medical Team Composition

Our present study highlighted the large imbalance between the number of women versus men on national medical teams. Only one out of five national medical team members were women. This gender disparity is in agreement with previous studies: [8, 25] Women represented approximately 10% of team physicians in the National Collegiate Athletic Association (with variation according to sports) [9, 10], 2.4% in the Mens’ National Basketball Association [11] and 28.6% in Women’s National Basketball Association [11]. O’ Reilly et al. [10] suggested some hypotheses to explain this disparity, which may be multifactorial, including “longer hours outside of clinical and surgical obligations, often requiring night and weekend time commitments”, and similar barriers than those of the gender imbalance in orthopaedic surgery (including e.g., lack of woman mentorship, different perception of expectations, work-life balance, constraints of traditional gender-based roles) [8, 25]. The fact that being a national medical team member implies traveling long times away from home and family could also play a role [8]. We can also not omit that this could be due to discrimination and the aim of the preservation of predominantly men medical team members.

In our present study, the gender imbalance differed within each profession, with a lower proportion of women among physiotherapists (19% vs. 24% among physicians). One potential explanation from anecdotal experience could be that a difference between physiotherapists and physicians may be due to travel commitments. A physiotherapist often travels to camps, small competitions as well as championships, whereas a physician (in some countries) may only travel to major championships. Therefore, overall travel commitments for a woman physician may be less of a barrier than for a woman physiotherapist.

### National Medical Team Composition Was Associated with the Team Performance

Our present correlation analysis showed that a higher number of athletes per national medical team member was associated with a lower number of medals per athlete. However, performance is multifactorial, our present study did not consider many confounders, and our results showed extensive heterogeneity among teams (Fig. 1). Health can be considered as one of several factors related to performance during international athletics championships [26–28]. The national medical team composition cannot causally determine the team’s and athletes’ performance, this represents only a factual result, for which we do not prefer to do any interpretation or any conclusion. As a factual result, it could maybe be used to orient the decision-making of the composition of national medical teams.

### Limitations

This was a retrospective analysis. This is a simple observational study from which we are not able to make recommendations. It is not possible to conclude causal relationships, and if the reported compositions were appropriate for athletes’ health management. In the European Athletics list of registered national medical team members, only two different categories were available: physicians without any information about their respective specialties and physiotherapists. For this latter category, it was not stated if these were physiotherapists as recognised by the profession, or other therapists (e.g., chiropractor, soft tissue therapist, osteopath), however, the category/term was chosen by the medical team members during the registration process. Future studies should increase the details and specificities collected about medical team member professions. The present results were representative of the European Athletics Championships, and their generalisation to other continents or sports is subject to caution. Also, only some potential factors that could play a role in the national medical team composition and team performance were included in the present analysis. Potential unmeasured confounding variables could have influenced the results. For example, there might be members of the national medical team who were involved in the preparation of the championships but did not travel with the team to the championships. In future studies, it would be of interest to include information regarding the national medical team’s composition out of competition. We had no information about the resources and economic costs that are needed to support the medical teams and/or the remuneration/compensation for medical providers, which can also play a role in the medical team composition, and thus can represent perspectives for future studies. Finally, we had no information regarding the preferences and needs of the athletes regarding the national medical teams’ composition.

### Practical Implications

The overall goal of this study was to help countries/teams prepare their national medical teams, regarding size and composition, for international athletics championships. Although the present study design does not allow us to make any recommendation, by reporting what has been done for the European Athletics championships during the 16 previous years it can be suggested that (i) when a country has more than ten athletes, most will have a national medical team, and (ii) defining the size of the national medical teams, according to the median values, a ratio of one physiotherapist for every 11 athletes and one physician for every 23 athletes could be taken into account. It is also noticeable to note that a lower number of athletes per national medical team member was associated with more medals per athlete. It is also important to promote the involvement of women health professionals in national medical teams since they can provide some specific benefits for athletes’ care management [5–8, 10, 25]. Decisions for medical team composition should thus be adapted according to each country’s context and specificities, athletes’ perceptions and requirements, and health professionals’ experiences.

## Conclusions

During the European Athletics championships over the 16 previous years, approximately two-thirds of countries had a national medical team, and among countries/teams with more than 10 registered athletes 98.7% had a national medical team. According to the median values, there were 8 athletes per medical team member, with large variability among countries. Only one out of five medical team members were women. Higher numbers of athletes per national medical team member were associated with lower numbers of medals per athlete. These results can serve as a basis for improving of medical coverage during these championships, with the overall goal of improving athletes’ health and performance.

## Electronic Supplementary Material

Below is the link to the electronic supplementary material.

Supplementary Material 1

## Data Availability

Data are available upon reasonable request. Requests for data sharing from appropriate researchers and entities will be considered on a case-by-case basis. Interested parties should contact the corresponding author Pascal Edouard (pascal.edouard@univ-st-etienne.fr).

## Abbreviations

EOC: European outdoor championships
EIC: European indoor championships
GDP: Gross domestic product per capita
IQR: Interquartile range
