# Supplementary material for: National Medical Teams during European Athletics Championships from 2009 To 2024: Composition, Gender Distribution, and Influence on Team Performance
Source: Sports Med Open. 2025 May 15;11:53. doi: 10.1186/s40798-025-00864-3 (PMC12081799; doi:10.1186/s40798-025-00864-3)
Supplement: Supplementary file 1 — Supplementary Material 1 [file 40798_2025_864_MOESM1_ESM.docx]

Sports Medicine – Open

Original research

**National medical teams during European Athletics championships from 2009 to 2024: Composition, gender distribution, and influence on team performance**

Pascal Edouard^1,2,3^, Spyridon Iatropoulos^1^, Karolina Velebova^4^, Ciara McCallion^5^, Marianna Kiss^6^, Pierre-Eddy Dandrieux^1,7^, Pedro Branco^3^, Jane Thornton^8,9^, Karsten Hollander^10^

From:

^1^ Inter-university Laboratory of Human Movement Science (LIBM EA 7424), University of Lyon, University Jean Monnet, F-42023. Saint Etienne, France

^2^ Department of Clinical and Exercise Physiology, Sports Medicine Unit, University Hospital of Saint-Etienne, Faculty of Medicine, Saint-Etienne, France

^3^ European Athletics Medical & Anti Doping Commission, European Athletics Association (EAA), Lausanne, Switzerland

^4^ Body Solution Clinic, Prague, Czech Track and Field Association, Czech Olympic Team

^5^ Athletics Ireland, Northwood Court, Northwood Business Campus, Satrym Dublin, Ireland

^6^ National Institute for Sports Medicine, Budapest, Hungary

^7^ Mines Saint-Etienne, Univ Lyon, Univ Jean Monnet, INSERM, U 1059 Sainbiose, Centre CIS, F-42023 Saint-Etienne France

^8^ Medical and Scientific Department, International Olympic Committee, Lausanne, Switzerland

^9^ Western Centre for Public Health & Family Medicine, Schulich School of Medicine & Dentistry, Western University, London, Ontario, Canada

^10^ Institute of Interdisciplinary Exercise Science and Sports Medicine, MSH Medical School Hamburg, Hamburg, Germany

**Correspondence to**

Pascal Edouard, MD PhD, Department of Clinical and Exercise Physiology, Sports Medicine Unit, IRMIS, Campus Santé Innovations, University Hospital of Saint-Etienne, 42 055 Saint-Etienne cedex 2, France. Tel.: +33 674 574 691; Fax numbers: +33 477 127 229; E-mail: Pascal.Edouard42@gmail.com

Supplementary materials:

Number of supplementary data: 2 supplementary tables and 1 supplementary figure

**Supplementary Figure 2:** Associations between the national medical team size and the athletes’ team size (r=0.89; p<0.001).

**
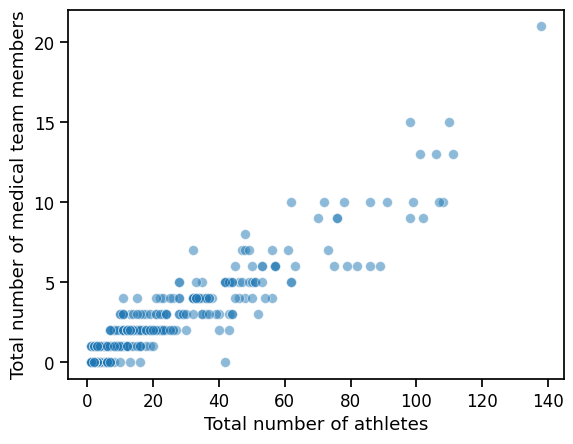
**

**Supplementary table 5: Spearman’s correlations between country-related characteristics and national medical teams’ characteristics**

|  | National medical team size | Ratio of registered athletes per medical team member | Profession disparity | Gender disparity |
| --- | --- | --- | --- | --- |
| Athletes’ team size | r=0.89; p<0.001 | r=0.54; p<0.001 | r=-0.26; p<0.001 | r=-0.17; p=0.01 |
| Total country population | r=0.70; p<0.001 | r=0.23; p<0.001 | r=-0.19; p=0.003 | r=-0.09; p=0.17 |
| Gross domestic product per capita (GDP) | r=0.04; p=0.56 | r=-0.05; p=0.48 | r=0.38; p<0.001 | r=-0.28; p<0.001 |
| Status of sports medicine specialty | r=0.08; p=0.23 | r=0.18; p=0.007 | r=-0.30; p<0.001 | r=0.12; p=0.06 |

**Supplementary table 6: Spearman’s correlations between national medical teams’ characteristics and team performance**

|  | Ratio of registered athletes per medical team member | Profession disparity | Gender disparity |
| --- | --- | --- | --- |
| Number of medals per athlete | r=-0.33; p<0.001 | r=-0.04; p=0.64 | r=0.07; p=0.38 |
